# Factors specifying sex determination in maize

**DOI:** 10.1007/s00497-023-00485-4

**Published:** 2023-11-15

**Authors:** Cristina Guerrero-Méndez, María Jazmín Abraham-Juárez

**Affiliations:** Laboratorio Nacional de Genómica para la Biodiversidad (LANGEBIO), Unidad de Genómica Avanzada, Centro de Investigación y de Estudios Avanzados del Instituto Politécnico Nacional (CINVESTAV), 36821 Irapuato, Mexico

**Keywords:** Floral organs, Sex identity, Hormones, Feminized, Maize

## Abstract

Plant architecture is an important feature for agronomic performance in crops. In maize, which is a monoecious plant, separation of floral organs to produce specific gametes has been studied from different perspectives including genetic, biochemical and physiological. Maize mutants affected in floral organ development have been key to identifying genes, hormones and other factors like miRNAs important for sex determination. In this review, we describe floral organ formation in maize, representative mutants and genes identified with a function in establishing sexual identity either classified as feminizing or masculinizing, and its relationship with hormones associated with sexual organ identity as jasmonic acid, brassinosteroid and gibberellin. Finally, we discuss the challenges and scopes of future research in maize sex determination.

## Introduction

Reproduction is the process by which all the species may preserve, copy and transmit genes; hence, it may be considered one of the most important aspects in the life of most organisms. In plants, the different forms of reproduction have favored an adequate genetic variability to achieve their survival and evolution. Between the different kinds of evolutionary strategies to increase reproduction efficiency is the development of sexual organs (De Craene 2018).

The discovery of reproductive organs in plants is attributed to the German physician and botanist Rudolf Jakob Camerarius, who wrote a letter about sex in plants which was published in 1694 (Žárský and Tupý 1995) starting a scientific revolution to explain how mating in plants works. For reproduction in the plant kingdom different molecular mechanisms have arisen naturally, leading to anatomical diversity. This diversity is intended to promote “outcrossing” which will consequently lead to heterogeneity as well as to generate genetic variability, assuming the adaptability of organisms (Dellaporta and Calderon-Urrea 1993).

There is a broad diversity of modalities for reproduction in plants. For instance, a plant can be bisexual, unisexual, monoecious, or dioecious, among others. Bisexual flowers are the most common in plants; according to Yampolsky and Yampolsky (1922), 75% of the flowering species are bisexual, while having unisexual flowers is not so common in nature; since only around 4% are monoecious, producing flowers considered “incomplete” because they are only male or female but those flowers are both in the same plant; finally, around 7% are dioecious, namely unisexual organisms producing only female or male flowers (Ashton 1969). Sexual determination in plants has evolved independently in many different species throughout history (Chuck 2010), so, it cannot be considered that there is a single gene network that determines sex identity in flowers, but that there are a whole set of factors involved, such as the environment, hormones and genes (Dellaporta and Calderon-Urrea 1994).

## The maize flower

**Fig. 1:**
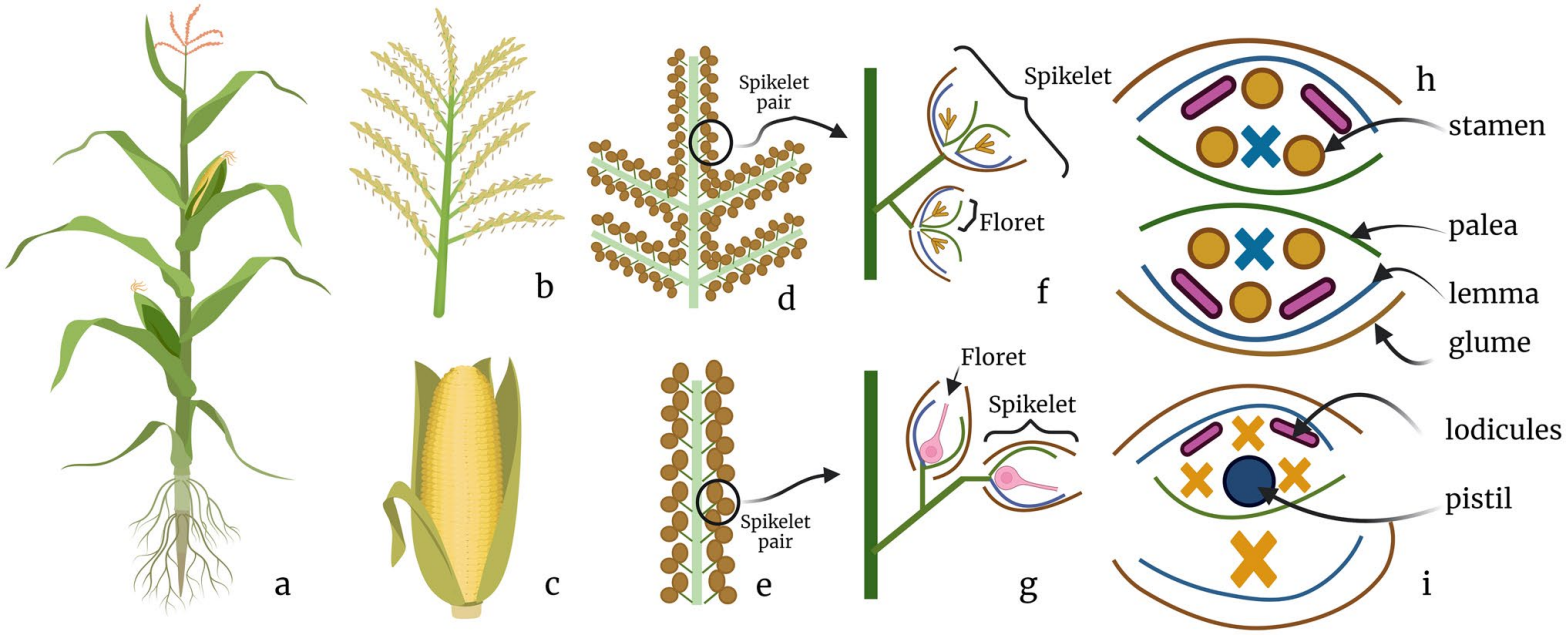
Floral organ development in a maize plant. **a** Adult maize plant in reproductive stage. **b** Mature tassel. **c** Mature ear. **d**, **e** Male and female spikelet pairs. **f**, **g** Male and female florets, respectively; the first ones are double and the second ones are single. **h** Male flower development with pistil suppression (labeled with blue “x”). **i** Female flower development with anther suppression (labeled with yellow “x”)

*Zea mays* is considered a monoecious species, since it produces female inflorescences called “ears” and male inflorescences called “tassels”, in separate positions in the same plant (Cheng et al. 1983). Unisexuality in maize is caused by the abortion that occurs selectively in both floral organs, male and female (Fig. 1); in the male flower carpel development is suppressed, while in the female flower anthers are suppressed; in addition, in female inflorescences a half of all florets are aborted (Yang et al. 2021).

Female floral organs in maize are found on ears produced in the axil of leaves along the stem, distributed in an interleaved way, while male flowers are located at the apex of the plant (Dellaporta and Calderon-Urrea 1994). This growth pattern and characteristic morphology to each one makes each type of flower easily distinguishable. Some maize inbred lines form “tillers” (smaller plants originating from the same main stem) that may follow the mother plant pattern and produce tassel and ears, tillers are dependents on genetics, hormones and photoperiod (Cheng et al. 1983). At genetic level it is well known the function of the transcription factor *Tb1* (*Teosinte branched 1*) negatively regulating outgrowth of tillers by activation of *gt1* (*grassy tillers 1*) and positively regulating hormones like abscisic acid and jasmonic acid, so, *tb1* and *gt1* mutants have more tillers (Dong et al. 2019; Whipple et al. 2011). However, the molecular basis to form tillers depending of photoperiod has been little explored. Some reports relating tillering with light quality (red/far red ratio) showed that high ratio of far red light perceived by a PHYTOCHROME B (PHYB) activates *Tb1* expression, consequently affecting the downstream pathways leading to tiller formation and so increasing apical dominance (Kebrom et al. 2006; Dong et al. 2019).

Despite the fact that there is an important anatomical difference in tassels and ears at the adult stage of development, both male and female floral meristems are very similar in morphology showing initially bisexual flowers, but it is not identical, since it has been reported a difference in meristem growth rate and branching (Cheng et al. 1983; Irish and Nelson 1989). Throughout development at the morphological level, meristems gradually enlarge to shape the inflorescence. Unlike Arabidopsis or other model species where inflorescence meristem (IM) directly produces floral meristems (FM), in grasses IM first produces higher-order meristems in this order: IM forms spikelet pair meristems (SPM), which produce two spikelet meristems (SM), then, SM give rise to lower and upper floral meristems (LFM/PFM) (Yang et al. 2021). In male inflorescence, elongation produces branching to give place the tassel architecture, which does not occur in ears. Both male and female flowers start forming two subtended glumes which protect floral meristems, and then a variety of organs are formed from floral meristems including lemma, palea, stamens and gynoecium (Fig. 1h, i). At the early stage of development, both flowers are considered bisexual; however, as development progresses at the tassel primordia, gynoecium cells become highly vacuolated and end up degenerating, while anthers cells continue cell division until reaching sexual maturity; also in some genetic backgrounds red and purple flavonoids are synthesized and stored at this tissue (Dellaporta and Calderon-Urrea 1994). Reciprocally, at the ears primordia, anther cells arrest and abort, while carpel cells division continues to form mature female organs (Yang et al. 2021). It has been proposed that sex determination by programmed cell death (PCD) in maize occurs at a late stage, probably after expression of homeotic genes that specify floral organ identity (Chuck 2010). In this way, upon reaching reproductive maturity there will be a clear distinction between the male and female flowers.

## Maize mutants affected in sexual determination and their relationship with hormones

Over nearly a century, a number of maize mutants have been described, which made it possible to recapitulate the molecular history of the genes that have participated in sex flower determination. These mutants have been identified because they present phenotypes in which there is no carpel or anther abortion. We have divided them in two sections: feminized and masculinized.

## Feminized mutants

A considerable number of mutants have been described as feminized, namely, that do not present carpel abortion in tassels. Broadly studied feminized mutants are the ones affected in genes related to Jasmonic acid (JA) pathway. Representative examples are *tasselseed* (*ts*) mutants, which have been extensively studied for the last 3 decades, the classic phenotype in *ts* mutants is a transformation of male to female flowers. By using double *ts* mutants, they could be classified in two classes: I and II, in function of the observed genetic interactions (Irish et al. 1994). Class I mutants show a feminized phenotype, while class II shows a more complex phenotype, where in addition to feminization they have an irregular branching pattern in inflorescences. As a first approach to describe genetic interactions, it was proposed that mutations were acting epistatically; however, then double-mutant phenotypes suggested a synergy between both classes (Irish 1997). These phenotypes are shown as sterile and highly branched inflorescences, so, in addition to carpel suppression in tassels, *ts* genes may have a role either directly or indirectly in inflorescence organogenesis and branching regulation (Irish 1997).

### Class I

This class includes the recessive *ts1* and *ts2* mutants, and the dominant *Ts3* and *Ts5* mutants, which do not suppress carpels in the tassels; as a consequence, they show an abnormal feminized phenotype, in some genetic backgrounds being able to form fertile bisexual flowers that self-pollinate producing seeds in tassels (Emerson 1920; Nickerson and Dale 1955; Irish et al. 1994; Lunde et al. 2019).

The *ts1* gene encodes a lipoxygenase called ZmLOX8, which functions directly in the jasmonic acid (JA) biosynthesis pathway (Acosta et al. 2009). It was suggested that this enzyme is involved in JA biosynthesis, based on observations by Acosta et al. about the drastic low level of endogenous JA in *ts1* mutants. Accordingly, when JA is exogenously applied to *ts1* tassels, the wild-type phenotype is recovered. Based on those experiments, a relationship between JA and male flower development in maize was proposed. *ts2* encodes an alcohol dehydrogenase enzyme, which seems to be involved in metabolism of steroidal molecules or in metabolism of GA-like compounds (DeLong et al. 1993). To test the ability of the TS2 protein to bind hormones, Wu et al. (2007) explored the binding between TS2 and JA or GA; however, no binding was detected. Apparently *dwarf* mutants (affected in GA biosynthesis) and Class I *tasselseed* mutants function in separate pathways, but it is still questionable how the GA exogenous application rescues the *ts2* mutant (Wu et al. 2007).

Other Class I *ts* mutants include some dominants like *Ts3* and *Ts5*, which show similar phenotypes to *ts1* and *ts2*, but not so severe (Nickerson and Dale 1955; Neuffer et al. 1997). The *Ts5* mutant is considered a loss-of-function dominant mutant affected in the JA biosynthetic pathway, *Ts5* overexpresses the gene *CYP94B1* which is wound inducible and inactivates the JA precursor jasmonoyl-L-isoleucine (JA-Ile), so *Ts5* tassels show lower JA and JA-Ile than tassels in wild-type plants. Based on those observations it was proposed that the *Ts5* phenotype results from JA signaling pathway disruption, and that this hormonal imbalance affects maize monoecious development (Lunde et al. 2019). Finally, the dominant *Ts3* mutant is waiting for a better characterization at the phenotypic and molecular level; its feminized phenotype was described by Nickerson and Dale (1955); however, detailed analyses are still needed.

### Class II

In this class, the *ts4* and *Ts6* mutants are included; mutants in this class besides being feminized show an atypical branching pattern both in ears and tassels. This kind of mutations affects the fate of reproductive meristems; *ts4* mutants show an indeterminate spikelet meristem, pistils are not suppressed in tassels, and male flowers do not develop, resulting in complete feminization (Chuck et al. 2007). *Ts6* mutants are delayed in the conversion of reproductive meristems; in addition, the atypical branching is suppressed at the base of tassels allowing forming normal spikelets (Irish 1997). On the other hand, the dominant mutant *Ts6* shows indeterminate floral meristem (Irish 1997), and consistently with this phenotype it mis-expresses the *Knotted1* homeobox gene (Jackson et al. 1994), indicating that there is a delay in meristem differentiation. The *Ts6* mutation site affects the *ts4* binding site, and *ts4* encodes a microRNA (miR172), subsequently affecting transcription factors binding APETALA2, necessary for floral meristem determination (Chuck et al. 2007; Banks 2008). Since *Ts6* is a dominant mutant, it has been hypothesized that *Ts6* functions mis-expressing a factor which causes prolonged meristem activity (Chuck 2010).

On the other hand, the *nana plant 1 (na1)* and the *required to maintain repression 6 (rmr6)* mutants have a phenotype similar to the *tasselseed* group, with carpel growth in male flowers (Parkinson et al. 2007; Hartwig et al. 2011; Li and Liu 2016). *na1* has a very low endogenous brassinosteroid (BR) level; analysis of *na1* mutant identified a loss-of-function mutation in a DET2 homolog gene, which functions in BR biosynthesis; in consequence, *na1* shows an accumulation of the DET2 substrate (24R)-24-methyl- cholest-4-en-3-one and a decrease in BR metabolites. Accordingly, exogenous application of BR inhibitors in wild-type maize plants produces the phenotype of *na1* (Hartwig et al. 2011); these experiments showed the relationship of NA1 with BR biosynthesis. The *rmr6* mutant possesses a tassel with female characters, forming bisexual fertile flowers and seed production, unlike the *tasselseed* and *na1* mutants (Hollick et al. 2005; Parkinson et al. 2007). *rmr6* is also affected in internode elongation with reduced apical internodes, genetic analysis and association with factors involved in transition from vegetative to reproductive stage of development has related *rmr6* with GA and Auxin pathways (Li et al. 2023). However, additional analyses are required to decipher the precise molecular function of *Rmr6*.

Another recently reported feminized maize mutant is *gt1;ra3* (Klein et al. 2022); this double mutant was originated from a *gt1* (*grassy tillers 1*) mutant enhancer screening to identify genes that together with the *gt1* transcription factor are involved in carpel suppression in tassels; from this screen, *ra3* (*ramosa 3*) was identified. *gt1;ra3* is affected in carpel suppression due to a mutation in the transcription factor GRASSY TILLERS1 (GT1) and in the trehalose-6-phosphate phosphatase (TPP) gene encoding the RAMOSA 3 protein, producing carpel growth in tassels and therefore feminized male flowers (Klein et al. 2022). *gt1* mutant itself has a weak carpel suppression phenotype and it was identified by tiller formation and its genetic relationship with *Tb1* (Whipple et al. 2011; Dong et al. 2019). In addition, the function of GT1 has been related to light; it was reported to be induced by shade conditions, controlling pistil growth inhibition by acting downstream of the PCD signal from *TS2* (Whipple et al. 2011; Bartlett et al. 2015). Transcriptomic analysis of *gt1;ra3* in feminized tassel development showed 73 genes with function related to carpel suppression, for example, PCD, ROS, proteolysis, sugar metabolism and ABA and GA response related genes (Klein et al. 2022). All together these findings suggest a key role of hormones and light in floral organ identity; however, in maize there is still a long way to go to understand the relationship between light quality, hormonal balance and sex determination.

## Masculinized mutants

In addition to JA and BR, GA (Gibberellin) is another plant hormone that has been related to sex determination in maize, the clearest example is the exogenous application of GA in wild-type tassels causing lack of carpel suppression in male flowers (Nickerson 1959). A number of reports have shown a direct relationship of high GA level with feminization and low GA level with masculinization. There is a group of mutants affected in genes encoding enzymes of the gibberellin biosynthesis pathway; they are the *dwarf* (*d*) *d1*, *d2*, *d3*, *d5* recessive mutants and the *Dwarf* (*D*) *D8*, *D9* dominant mutants (Winkler and Freeling 1994; Fujioka et al. 1988). These mutants have been classified as GA-sensitive and GA-insensitive, as the name suggests, GA-sensitive mutants are able to recover the wild-type phenotype by exogenous application of GA, but GA-insensitive mutants do not (Phinney 1984). Some of these mutants have a low level of GA due to defects in different steps of the synthesis, for instance, *d3* which encodes the GA 3-oxidase enzyme that catalyzes the final step of the GA synthesis (Winkler and Helentjaris 1995; Chen et al. 2014).

On the other hand, *D8* and *D9* mutants, categorized as GA-insensitive, are mutants affected in genes encoding DELLA (aspartic acid–glutamic acid–leucine–leucine–alanine) domain transcription factors (Peng et al. 1999; Lawit et al. 2010), which act as master repressors of the GA signaling pathway. The *D8* mutant does not respond to GA and has been proposed that the *dwarf plant 8* (*d8*) may be a receptor of GA (Fujioka et al. 1988). Peng et al. (1999) showed that *D8* has a deletion that removes four amino acids from the DELLA domain, suggesting that this is the reason for its insensitivity to GA.

Unlike the *D8* mutant, it took at least a decade before *D9* began to be properly characterized. The first report of the mutant was by Winkler and Freeling (1994), who confirmed that it is a paralog of the affected gene in the *D8* mutant, although both genes are located in different chromosomes, in *D9* a DELLA protein is also affected, with point substitutions in a pair of amino acids. Dwarfism also occurs in *D9* mutant, and accordingly to proposed function, late flowering is observed when this gene is overexpressed (Lawit et al. 2010).

Inflorescence phenotype in *Dwarf* mutants either recessives (*d*) or dominants (*D*) is related to reduction of GA level, and it shows different degrees of a masculinizing phenotype in ears with anther development in ear florets, which produces upper female ear florets with both anthers and pistils, showing bisexual flowers and lower ear florets with androecium identity (Best and Dilkes 2022). The phenotype in tassel anthers is mostly like in wild-type plants, with normal anther development but in some cases with compact tassels (Best et al. 2016).

Another masculinized mutant is the *Anther-ear 1* (*An1*), which was first described by Emerson and Emerson (1922); it has an affected phenotype in plant height although not as severe as the GA-insensitive mutants; in addition, it presents delayed sexual maturity and development of bisexual flowers in the ears location, with anther development in the proximal and distal female spikelet florets (Bensen et al. 1995). This mutant is sensitive to GA, since the affected gene is involved in the synthesis of ent-kaurene, which is the first intermediate in the GA biosynthetic pathway. Like other previously described mutants, this one shows a positive and regenerative response to monoecious flowering with exogenous application of GA (Bensen et al. 1995). Overall, it can be assumed that GA biosynthesis and signaling are necessary for the proper formation of both male as female flowers in maize.

The last one mutant affected in floral organ development described here is the recessive *silkless1* (*sk1*); it has normal tassel development, but completely sterile ear flowers by the absence of pistils and some anther persistence in ear florets (Hayward et al. 2016; Best and Dilkes 2023). The affected gene in *sk1* encodes a UDP-glycosyltransferase that regulates endogenous JA level by inactivation of JA via conjugation; thus, *sk1* mutant shows high level of JA (Hayward et al. 2016). According to this function, when the *sk1* gene was constitutively expressed in transgenic plants, all pistils in female flowers were completely developed and even produced complete feminization in tassels demonstrating that *sk1* gain of function blocks the accumulation of jasmonates and *sk1* lack of function produces accumulation of JA in maize florets (Hayward et al. 2016). In addition, the androecious flower phenotype in *sk1* is enhanced in the double mutant *d5 sk1*, showing an interaction of GA and JA pathways to establish floral organ identity in maize (Best and Dilkes 2023).

**Fig. 2:**
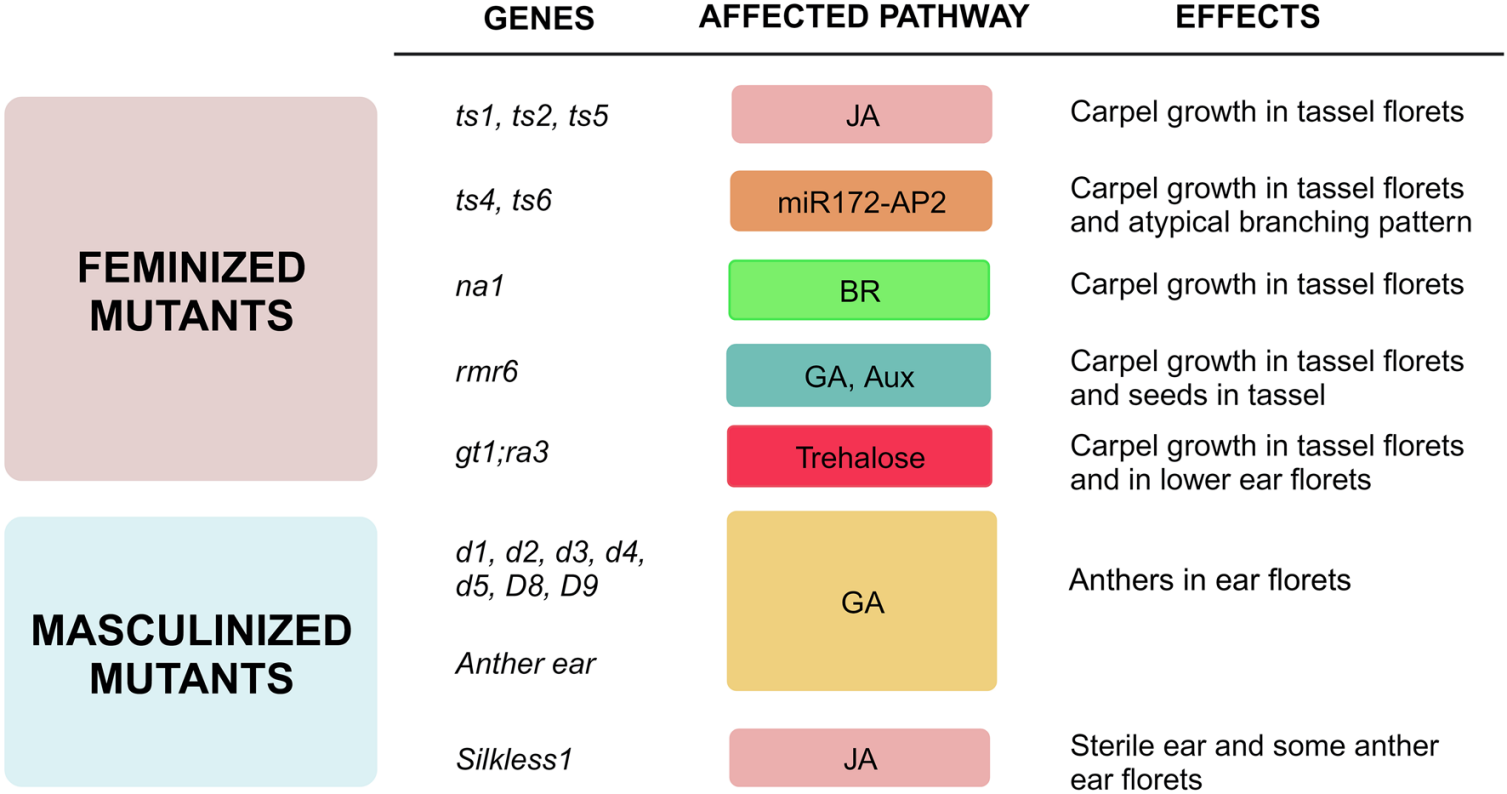
Summary of feminized and masculinized mutants, affected genes, related pathways and effects on phenotype

In summary, in maize there are multiple pathways relating genes and hormones converging in different developmental stages to determine floral identity and related phenotypes to this process; Fig. 2 shows representative mutants, genes and pathways where they are working as well as effects in phenotype when their function is disrupted.

## Challenges and scopes of research in maize sex determination

Advances in genomics and transcriptomics either using organ tissues or single-cell ‘omics’ are giving light to understand biological processes under studied before the development of high resolution technologies in maize; thanks to such technologies, it is possible to have transcriptome atlas of the maize inflorescences through development (Satterlee et al. 2020; Xu et al. 2021). In addition, the development of new gene-editing technologies (Liu et al. 2021) is increasing exponentially the amount of allelic variants either in regulatory or in coding sequences for study of genetic determinants important in maize sex identity. On the other hand, metabolomic and proteomic approaches are still a challenge in grasses, since the amount of secondary metabolites in different tissues frequently interfere with getting reproducible results based on analyte quantification (Walley et al. 2016). However, with the development of technologies increasingly sensitive these tools will be as used as in model dicot species. With the increasing data from single-cell RNAseq (Satterlee et al. 2020; Xu et al. 2021) and interactomics (Wu et al. 2019; Abraham-Juárez et al. 2020; Han et al. 2023), it will be possible to correlate gene expression, co-expression pathways and interacting networks with specific biological functions such as feminizing or masculinizing gene functions in inflorescences and their relationship with hormonal pathways. Maize is a species of particular interest to understand and manipulate outcrossing on a large scale in the field, during production of hybrid lines in agriculture; also it is an excellent model to study gene evolution in grasses, so, understanding how organ identity is established to form floral structures is of huge interest in this species.

## Conclusion and perspectives

Maize is an important model species to study outcrossing mechanisms in grasses. Thanks to the knowledge generated regarding developmental biology and reproduction in maize, identification and use of natural and induced mutants have been possible. In turn, characterization of developmental mutants has been key to identify genes and pathways with functions in sex determination. Very importantly, the growing genomic, transcriptomic, metabolomic and recently proteomic information in maize together with development of new gene-editing technologies is giving light in the understanding of molecular processes involved in sexual organ identity establishment. However, given the large number of factors involved in sex determination not only in maize, but in different species, elucidation of molecular mechanisms in this process remains as a challenge in plant biology. Environmental factors and their relationship with the genetic ones are among the least studied, and due to climate change, these are attracting more and more interest from researchers in the developmental biology field especially in species of agronomic interest. Therefore, multidisciplinary approaches are necessary to elucidate the molecular processes that are key to determine cell identity and that could be manipulated to improve the performance of cultivable plants such as maize.
